# Identification of Copy Number Variation in Domestic Chicken Using Whole-Genome Sequencing Reveals Evidence of Selection in the Genome

**DOI:** 10.3390/ani9100809

**Published:** 2019-10-15

**Authors:** Donghyeok Seol, Byung June Ko, Bongsang Kim, Han-Ha Chai, Dajeong Lim, Heebal Kim

**Affiliations:** 1 Department of Agricultural Biotechnology and Research Institute of Agriculture and Life Sciences, Seoul National University, 1 Gwanak-ro, Gwanak-gu, Seoul 08826, Korea; sdh1621@snu.ac.kr (D.S.); kobanga@snu.ac.kr (B.J.K.); babybird93@snu.ac.kr (B.K.); 2 C&K Genomics, 26 Beonbwon-ro, Songpa-gu, Seoul 05836, Korea; 3 Animal Genomics & Bioinformatics Division, National Institute of Animal Science, RDA 1500, Kongjiwipatiwi-ro, Iso-myeon, Wanju-gun, Jeollabuk-do 55365, Korea; hanha@korea.kr (H.-H.C.); lim.dj@korea.kr (D.L.)

**Keywords:** CNV, Cornish, Rhode Island Red, White Leghorn, broiler, layer, economic trait, population differentiation, domestic animal, CNVnator

## Abstract

**Simple Summary:**

Chickens have been bred for meat and egg production as a source of animal protein. With the increase of productivity as the main purpose of domestication, factors such as metabolism and immunity were boosted, which are detectable signs of selection on the genome. This study focused on copy number variation (CNV) to find evidence of domestication on the genome. CNV was detected from whole-genome sequencing of 65 chickens including Red Jungle Fowl, broilers, and layers. After that, CNV region, the overlapping region of CNV between individuals, was made to identify which genomic regions showed copy number differentiation. The 663 domesticated-specific CNV regions were associated with various functions such as metabolism and organ development. Also, by performing population differentiation analyses such as clustering analysis and ANOVA test, we found that there are a lot of genomic regions with different copy number patterns between broilers and layers. This result indicates that different genetic variations can be found, depending on the purpose of artificial selection and provides considerations for future animal breeding.

**Abstract:**

Copy number variation (CNV) has great significance both functionally and evolutionally. Various CNV studies are in progress to find the cause of human disease and to understand the population structure of livestock. Recent advances in next-generation sequencing (NGS) technology have made CNV detection more reliable and accurate at whole-genome level. However, there is a lack of CNV studies on chickens using NGS. Therefore, we obtained whole-genome sequencing data of 65 chickens including Red Jungle Fowl, Cornish (broiler), Rhode Island Red (hybrid), and White Leghorn (layer) from the public databases for CNV region (CNVR) detection. Using CNVnator, a read-depth based software, a total of 663 domesticated-specific CNVRs were identified across autosomes. Gene ontology analysis of genes annotated in CNVRs showed that mainly enriched terms involved in organ development, metabolism, and immune regulation. Population analysis revealed that CN and RIR are closer to each other than WL, and many genes (*LOC772271*, *OR52R1*, *RD3*, *ADH6*, *TLR2B*, *PRSS2*, *TPK1*, *POPDC3*, etc.) with different copy numbers between breeds found. In conclusion, this study has helped to understand the genetic characteristics of domestic chickens at CNV level, which may provide useful information for the development of breeding systems in chickens.

## 1. Introduction

Structural variation refers to the variation above 1 kb in the genome including copy number variation (CNV), translocation, and inversion [1]. CNV is a genomic region that shows quantitative variants when compared to the reference genome and is comprised of deletion and duplication regarding copy number [2]. With the alteration of gene structure or dosage [3], CNV shows larger variation compared to small-scale variation such as single nucleotide polymorphism (SNP). This means that CNV could have a significant impact on the phenotype of an individual [4]. Indeed, CNV has great impact both functionally and evolutionally [5,6] and has been studied on a variety of animals such as humans [7,8,9], cattle [10,11,12], pigs [13,14,15], chickens [16,17,18], goats [19], dogs [20], cats [21], etc. Recent studies have shown the importance of CNV [22] regarding the relations to human diseases (cancer [23,24], schizophrenia [25], depression [26]) and the major traits of livestock [27,28,29].

Chicken is not only a major source of protein providing meat and eggs [30] but also has great value to be utilized as bioreactors [31] and animal models of disease [32]. Therefore, various studies from the genomic point of view have been conducted to develop economic traits and reveal the mechanisms underlying phenotypic variation [33]. Beginning with avian evolution study through the comparative genomics approach between chickens and turkeys [34], CNV studies have several interesting findings such as identification of CNVs related to pea-comb [35] or muscle development [17] and breed-specific CNVs detected on the population-level [16,36].

Identification of CNV was usually processed through two types of microarray; array-based comparative genomic hybridization and SNP array. But now next-generation sequencing (NGS)based method is preferred for the following reasons: Microarray-based method cannot identify 1) small CNV, and 2) CNV at genome-wide level due to low probe density. Also, 3) the price of whole-genome sequencing is more affordable due to the development of NGS technology [37]. Although NGS is more expensive and requires more effort while used for diagnostic tests [38], it has more benefits; it is applicable for various analysis other than CNV, can be detected in poorly annotated regions, and other researchers’ data can be used as a control. Although NGS-based identification has been widely used on various animals, it has not been used much on chickens [39,40,41,42,43]. Existing studies also have limitations regarding analyzing breed-specific CNV region (CNVR) due to small sample sizes of 2–12 chickens. Kerstens et al. analyzed 100 chickens (25 per breed) using the reduced representation library only sequencing the regions around the restriction site which led to the limitations with identification of CNV compared to individual sequencing that uses full genetic information [39,44].

The goal of this study is to understand the phenotypic characteristics of commercial chicken breeds at the CNV-level with an NGS-based method, especially on the artificial selection during domestication. We used CNVnator [45], a widely used read-depth based CNV detection program. CNVs were identified on three breeds naturalized in South Korea [46]: Cornish (CN; broiler), White Leghorn (WL; layer), and Rhode Island Red (RIR; hybrid) [30], 20 per breed, a total of 60 chickens by whole-genome sequencing with approximately 10× depth from the public database. The identified CNVRs were compared with five Red Jungle Fowl (RJF; Gallus gallus) to distinguish CNVRs related to domestication. Finally, through functional annotation, we confirmed which gene ontology (GO) terms were enriched. Our findings will deepen the understanding of the breed-specific physiology and characteristics on a genome-wide level as the basis for future breeding systems.

## 2. Materials and Methods

### 2.1. Collection of Chicken Whole-Genome Sequencing Data

Twenty samples each from CN, RIR, and WL were downloaded (60 total samples), from the National Agricultural Biotechnology Information Center (NABIC; https://www.nabic.rda.go.kr) public database [47]. The project name is ‘Construction of standard genome maps of Korean native chicken’ and the accession number for the CN sample are NN-0991-000001 to NN-0991-000020, the RIR NN-0991-000081 to NN-0991-000100, and the WL NN-0991-000101 to NN-0991-000120.

Five RJF samples were downloaded from NCBI Sequence Read Archive (SRA; https://www.ncbi.nlm.nih.gov/sra) and were used as the control group. The experiment numbers for the five RJF chickens are SRX408166 [48], SRX511213, SRX511214, SRX511216, and SRX511217 [49].

All the data were sequenced on an Illumina HiSeq 2000 sequencer (Illumina Inc.) with 100bp paired-end reads.

### 2.2. Sequence Alignment to Reference Genome

The adapters of sequencing reads were removed by Trimmomatic-0.36 [50], and trimming was also performed where the Phred-score at the end of the read was less than 20 (TRAILING:20). Trimmed reads were mapped to galGal5 (Gallus gallus) assembly of the chicken genome using the bowtie2-2.3.3.1 [51] with the default option. PCR duplicates that could affect the CNV analysis were removed by using Picard-2.9.2 (https://broadinstitute.github.io/picard/) with Markduplicates. Finally, Genome Analysis Toolkit-3.3.0 [52] was used to realign reads for correcting errors caused by InDels.

### 2.3. CNV and CNVR Detection

CNVnator-v0.4 software [45], a read-depth based method, was used for CNV prediction relative to the galGal5 reference assembly. Since the average depth of the realigned bam files was about 10×, the optimal bin size was unified to 300bp for all individuals. The CNV calls were filtered with p-value <0.001, q0 (zero mapping quality) <0.5, and size >1 kb. To compare the copy number between breeds, the estimated copy number for each region was calculated using the ‘-genotype’ option of CNVnator.

CNVRs were obtained using ‘CNV_overlap.py’ script on GitHub (https://github.com/bjtrost/TCAG-WGS-CNV-workflow) [53], and only CNVRs found in three or more individuals of any breed were used for downstream analysis to remove false-positive results [54].

### 2.4. CNVR Annotation

The list of chicken autosome galGal5 genes was downloaded from NCBI, and the gene annotation was performed using the ‘annotatePeak’ command of the Bioconductor package ChIPseeker [55] with ‘addFlankGeneInfo = TRUE’ option. GO enrichment and Kyoto Encyclopedia of Genes and Genomes (KEGG) analysis were performed using Database for Annotation, Visualization and Integrated Discovery (DAVID; https://david.ncifcrf.gov/) [56,57] only on protein-coding genes. Biological process, cellular component, and molecular function were used as GO term categories, and the significance level was a p-value of 0.05. Also, the chicken’s quantitative trait loci (QTL) were downloaded from the chicken QTLdb Release 38 (https://www.animalgenome.org/cgi-bin/QTLdb/GG/summary) [58] and compared with the identified CNVRs. We only considered QTLs reported in galGal5 version with confidence interval less than 5 Mb. We used Bedtools-v2.27.1 [59] ‘intersect’ command to detect which QTL overlaps with the identified CNVRs.

### 2.5. Breed Differentiation

We examined population differentiation with CNVRs found only in domesticated chickens using three methods; clustering analysis, V_ST_ statistic, and ANOVA test. First, clustering analysis was performed using pvclust R package [60]. During this process, a scoring matrix encoded as 0 or 1 was used according to the presence or absence of CNVRs identified for each individual. Approximately unbiased (AU) p-value and bootstrap probability (BP) value were obtained through 10,000 multiscale bootstrap resampling based on unweighted pair-group average method (UPGMA), and they were shown in red and green on the edges of the clustering. Second, the V_ST_ statistic was applied to detect regions with high differentiation between populations [61]. To better understand the effect on each breed, protein-coding genes annotated in CNVRs were used for V_ST_ calculation, instead of CNVRs. V_ST_ was calculated for protein-coding genes as V_ST_ = (V_T_ − V_S_) / V_T_, where V_T_ is the total variance in copy number between two populations and V_S_ is the average variance within each breed. Finally, ANOVA test was performed to detect differences in copy number of genes between breeds. The genes used for ANOVA were the same as for the V_ST_ analysis. Since the sample size for each breed was the same (20 samples per breed), Tukey’s HSD test for post-hoc analysis was selected. In addition, for some of the genes found to be significant in the ANOVA test, the depth of coverage was obtained at a specific bin size to see the part of the gene with different copy number pattern. Depth of coverage was obtained using Mosdepth-0.2.6 software (https://github.com/brentp/mosdepth) [62].

## 3. Results

### 3.1. Identification of CNVs and CNVRs

Statistics of read mapping and CNVs are shown in Table S1. Mapping sequencing data to galGal 5 assembly showed an average 96.21% mapping rate and 11.51× depth. Except for RJF, an average of 340 deletions and 120 duplications were detected on autosomes with an average size of 26.9 kb for deletion and 16.1 kb for duplication (Table 1).

**Figure 1:**
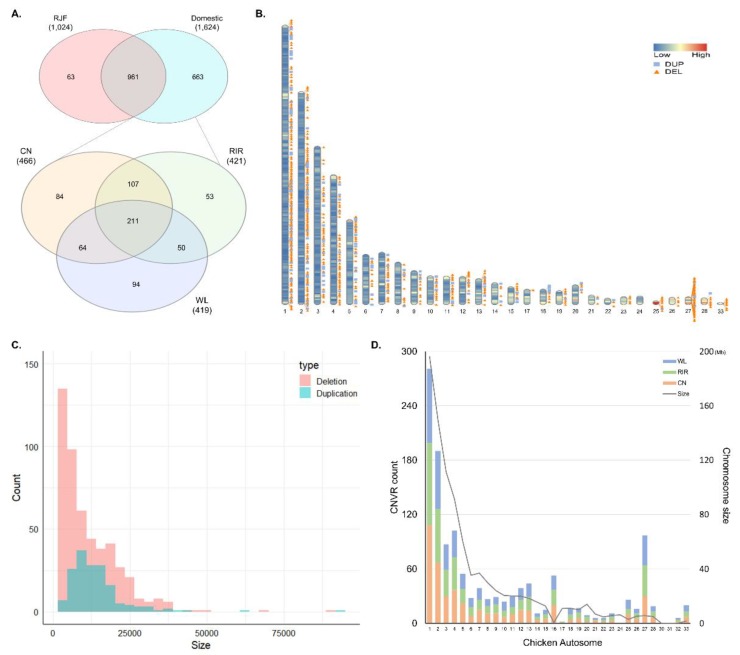
General description of the identified copy number variation regions (CNVRs). (**A**) Venn diagram for overlapping CNVRs between Red Jungle Fowl (RJF) and domesticated breeds (above), and among Cornish (CN), Rhode Island Red (RIR), and White Leghorn (WL) (below); (**B**) distribution of CNVRs on chromosome ideogram according to their state (deletion and duplication). This was drawn using Rideogram R package. The color painted on the chromosome represents the gene density; (**C**) distribution of CNVRs size by state; (**D**) chromosomal distribution of CNVRs for three chicken breeds and length of chicken autosome.

The overlapping regions of CNVs obtained using the TCAG-WGS-CNV workflow (https://github.com/bjtrost/TCAG-WGS-CNV-workflow) consisted of 3079 CNVRs with 2443 deletions sized from 1.2 kb to 514.8 kb and 636 duplications from 3 kb to 198.9 kb. Among the CNVRs combined from 65 samples, 1061 (34.5%) were detected in only one individual, 331 (10.8%) in two individuals, and 259 (8.4%) in three individuals. Of the 1687 CNVRs detected in three or more individuals, a total of 663 CNVRs were detected only in domesticated breeds (CN, RIR, and WL). Among the three breeds, the highest number of 466 CNVRs were detected in CN, followed by 421 in RIR and 419 in WL. A total of 211 CNVRs were detected in all three breeds. The number of breed-specific CNVRs was 84 in CN, 53 in RIR, and 94 in WL, respectively. CNVR detected in most individuals was duplication detected in 44 individuals, located on chr5 at 6.31–6.33 Mb, and second was deletion in 40 individuals, on chr1 at 11.42–11.44 Mb (Figure 1A; Table S2). Figure 1B shows the density of gene and distribution of all CNVRs we used for analysis on chromosome ideogram except for the microchromosome. We used CNVs with a minimum length of 1 kb in the analysis and the shortest CNVRs had a length of 1.8 kb. Four hundred and ninety-nine deletions ranged in size from 1.8 kb to 501 kb, with 275 deletions (55%) less than 10kb and 164 duplications from 3.9 kb to 92.1 kb with 81 duplications (50%) less than 12 kb (Figure 1C). The number of CNVRs on the chromosomes was not significantly different among the breeds. In general, the number of CNVRs detected was proportional to the chromosome size, but chromosomes such as 16 and 27 showed more CNVRs than longer chromosomes (Figure 1D).

### 3.2. Annotation of CNVR

We annotated genes corresponding to 663 CNVRs using NCBI Gallus gallus Annotation Release 103 (https://www.ncbi.nlm.nih.gov/genome/annotation_euk/Gallus_gallus/103/). When CNVR and gene overlap more than 1 bp, the relevant gene is annotated. If not, the closest gene is annotated. When there was another gene within 1 kb, it was additionally annotated because it may affect the expression of the gene. Six hundred and sixty-three CNVRs were annotated to a total of 856 genes, including 609 protein-coding genes, 200 lncRNAs, and 16 miRNAs. Four hundred and sixty CNVRs were annotated to only one gene, and up to 12 genes were annotated for duplication on chr27 at 578.7–641.7 kb (Table S3).

Functional enrichment analysis was performed on GO terms and KEGG pathways through DAVID with 491 protein-coding genes within 1 kb of CNVR. Twenty-two terms in biological process, five cellular components, seven molecular functions, and five pathways in KEGG pathway were significantly enriched (*p*-value < 0.05) and presented in Table S4. They were mainly involved in organ development, neuromodulation, immune regulation, and metabolism. In particular, in KEGG pathway category, pathways related to protein and carbohydrate metabolism (gga00051: Fructose and mannose metabolism, gga00350: Tyrosine metabolism, and gga00360: Phenylalanine metabolism) and xenobiotics metabolism (gga00982: Drug metabolism—cytochrome P450 and gga00980: Metabolism of xenobiotics by cytochrome P450) were significant.

The QTL reported in chicken QTLdb were downloaded to detect QTL associated with the identified CNVRs. A total of 432 QTLs reported as galGal 5.0 version and shorter than 5 Mb [16] were used (Production_QTL: 334, Physiology_QTL: 53, Health_QTL: 26, Exterior_QTL: 16, Reproduction_QTL: 3). The results showed that 165 QTLs overlapped more than 1 bp with some of the 663 CNVRs. The most detected category was Production_QTL, a total of 142 QTLs overlapped with CNVRs. The most detected QTL was breast muscle pH, followed by QTLs such as feed intake, ileum weight, abdominal fat weight, body weight and eggshell strength (Table S5).

### 3.3. Breed Differentiation of CNVR

**Figure 2:**
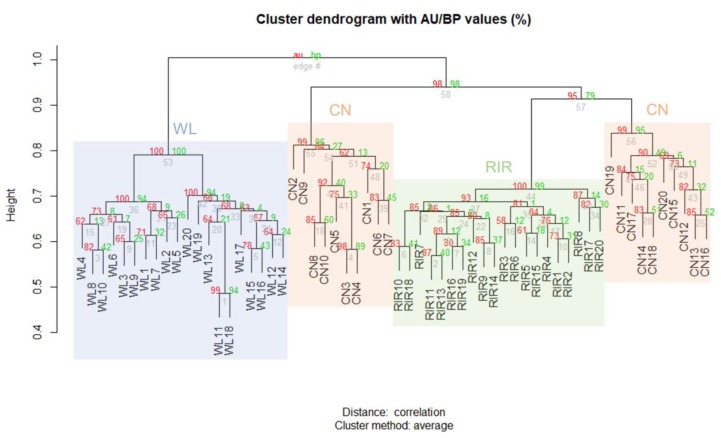
Hierarchical clustering analysis according to the presence or absence of CNVR.

Clustering analysis was performed on CNVRs identified only in domesticated breed through the result of each individual. Hierarchical clustering was created using the scoring matrix which represents the presence or absence of any CNVR in an individual, and the p-value was calculated by multiscale bootstrap resampling. The results are shown together with the AU p-value and BP value, and it was confirmed that they were significantly clustered within each breed (Figure 2).

**Figure 3:**
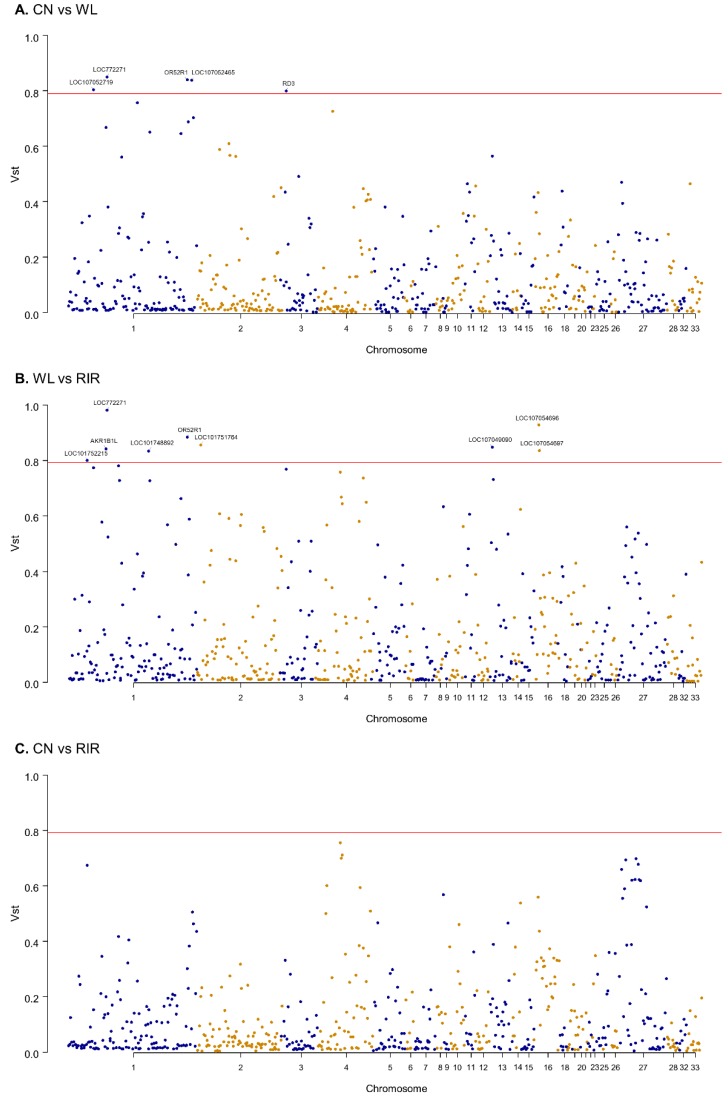
Manhattan plot of genome-wide genes’ V_ST_ value. (**A**) CN vs. WL; (**B**) WL vs. RIR; (**C**) CN vs. RIR. The red line represents 0.79 V_ST_.

We estimated V_ST_ statistic which is known to be similar to F_ST_ to examine population differentiation for CNVR [61]. V_ST_ value was calculated using the variance of copy number of 609 protein-coding genes annotated by CNVR to see population-specific selective pressure at gene-level. The average values of V_ST_ of the whole genes were 0.11 for ‘CN vs. WL’, 0.11 for ‘CN vs. RIR’, and 0.15 for ‘WL vs. RIR’ (Table S6). ‘WL vs. RIR’ showed the highest degree of differentiation, which is consistent with the above clustering result. To see genes with high differentiation between breeds, we examined genes with V_ST_ > 0.79, the top 98^th^ percentile. Five genes (*LOC772271*, *OR52R1*, *LOC107052719*, *LOC107052465*, *RD3*) in the ‘CN vs. WL’ pairs and nine genes (*LOC772271*, *OR52R1*, *LOC101752215*, *AKR1B1L*, *LOC101748892*, *LOC101751764*, *LOC107049090*, *LOC107054696*, *LOC107054697*) in ‘WL vs. RIR’ pairs exceeded the threshold and none of the ‘CN vs. RIR’ pairs exceeded the threshold. *LOC772271* (aldo-keto reductase family 1 member B10-like), which overlaps 1712 bp with CNVR41, and *OR52R1*, which is included in CNVR145 exceeded the threshold in both ‘CN vs. WL’ and ‘WL vs. RIR’ pairs (Figure 3).

**Figure 4:**
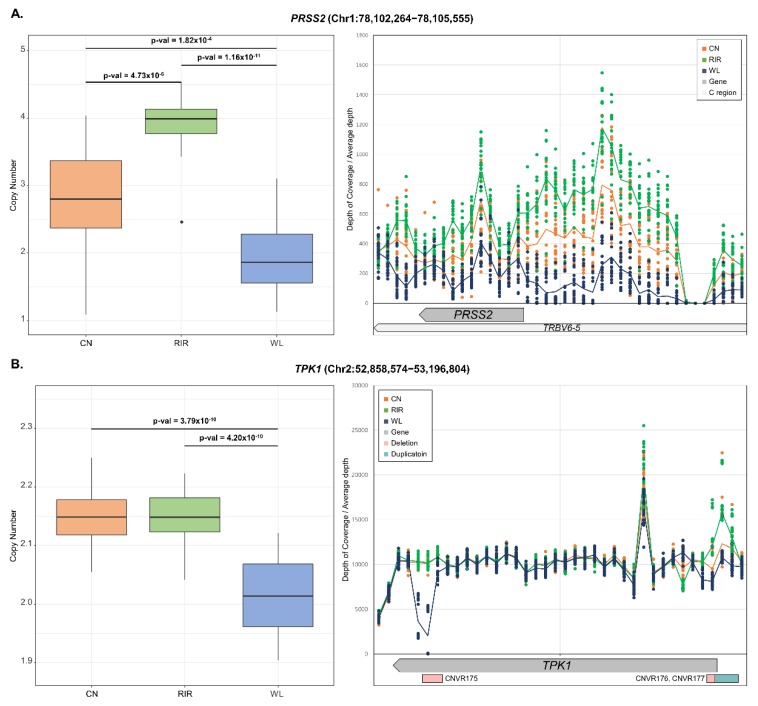
Estimated copy numbers of genes with different copy numbers between breeds and depth of coverage of gene region. The center line of the box represents the median and the line at the end of the box represents the first and third quartiles. Tukey’s HSD test was used to determine which breeds were significantly different (left). The depth of coverage of the region around the gene was extracted for a specific window size and divided by the total depth of each sequence file. The window size was determined by gene size. The scatter plot represents each individual and the line represents breed average (right). (**A**) *PRSS2*, window size: 300bp; (**B**) *TPK1*, window size: 10,000bp.

ANOVA was also performed to examine the differentiation of CN frequency and post-test was performed with Tukey’s test. As a result, 232 genes showed a statistically significant difference (*p*-value < 0.01) between breeds (Table S7). Even after Bonferroni correction (*p*-value < 0.01/609 = 1.64 × 10^−5^) and removal of the microchromosome with a chromosome size less than 20Mb, 63 genes remained significant. *LOC772271* (*p*-value < 3.55 × 10^−27^), and *OR52R1* (*p*-value < 1.16 × 10^−24^) mentioned in V_ST_ analysis were most significant, and many other genes also showed significance, such as *RD3* (*p*-value < 4.41 × 10^−20^), *ADH6* (*p*-value < 1.42 × 10^−17^), *TLR2B* (*p*-value < 7.6 × 10^−13^), and *POPDC3* (*p*-value < 6.16 × 10^−11^) (Figure S1). In most cases, only one out of the three breeds showed a difference in copy number. On the other hand, *PRSS2* (*p*-value < 8.68 × 10^−13^) showed different copy numbers among all three breeds (CN:RIR:WL = 1.5:2:1). We looked at the region around *PRSS2* through genome browser and found that *TRBV6-5* (biotype: C_region) overlapped with *PRSS2*. Checking the mapping read, it showed the same depth of coverage as *PRSS2* over the much larger region including *TRBV6-5* (Figure 4A). This region was also included in QTL with body temperature. There were cases where different copy number patterns were observed between breeds in one gene. For example, within *TPK1* gene (chr2: 52858574-53196804), WL showed deletion in CNVR175 (chr2: 52890601-52910100) and CNVR176 (chr2: 53185501-53190300), while CN and RIR with CNVR177 (chr2: 53190301-53209800) showed duplication (Figure 4B). Additionally, functional analysis was performed on significant genes through DAVID. As a result, pathways related to receptor signaling and metabolic activity were significantly enriched (Table 2).

## 4. Discussion

Using the NGS-based method to detect CNVs shows much better performance compared to the array-based method even in low-coverage situations [63]. However, since there is a possibility of false-positives [64,65], we used 20 individuals per breed with five RJF as a control group and only CNVRs identified in at least three individuals were used for analysis. Also, ANOVA test was performed and the actual depth of coverage was visualized for significant genes.

We identified a total of 1624 CNVRs for the CN, RIR, and WL, but only 633 (40.8%) remained after removing the commonly detected CNVRs from RJF. The reason for the high number of commonly detected CNVRs between domesticated breeds and RJF is probably that they share an evolutionary history before artificial selection. It is thought that genes which were important for survival such as nervous or immune system related genes were selected before domestication [66]. Even though microchromosomes such as chromosomes 16 and 27 are short in length, plenty of CNVRs have been detected in many previous studies [16,36,41]. CNVRs detected in various studies were combined into one CNVR on chromosome 16, which covers 99.95% of the full length of chromosome 16 [64]. This is because almost all genes mapped to chromosome 16 are immune-related, including major histocompatibility complex genes which have high levels of genetic diversity [67].

Chickens have been bred for a variety of purposes, including eggs, meat, and game [68]. For example, CN was bred for meat, WL for eggs, and RIR for both purposes [69]. The result of this breeding leads to unimaginable increases in productivity not found in RJF and is also found in various forms of variation on the genome [70]. Of course, this includes CNV, which affects productivity in a variety of ways, such as by deleting genes or changing gene dosage. The functional and QTL analysis resulted in enrichment of pathways of carbohydrate and protein related metabolism and organ development. This suggests that evidence of domestication can be confirmed at the CNV level.

Clustering and V_ST_ analyses showed that CN is closer to RIR than WL. In the V_ST_ results of ‘CN, RIR vs. WL’, two genes, *LOC772271* and *OR52R1*, were found to be common. *LOC772271* gene which is associated with fatty acid synthesis [71] was expressed more in the fast-growing line than in the slow-growing line [72] and has already reported a copy number difference between WL and ‘CN and RIR’ in the previous study [73]. *OR52R1* is an olfactory receptor gene (OR) and the copy number variable of OR was found in chickens as well as in pigs [74] and cattle [75]. Olfactory is associated with social communication of chickens. Especially laying hens may have high selection pressure on the copy number of OR due to housing conditions of sex-homogenous groups that are restricted from communicating with males [76]. In addition, various factors such as cognition of predators [77], daily activity pattern, habitat, and diet can act as a selective pressure on OR [78], indicating that copy number could vary between breeds.

ANOVA test showed differences in copy number between breeds in many genes. In *RD3* gene involved in photoreceptors [79], deletion occurred with CNVR261 in fifteen WL. Since light has a major effect on egg production [80,81], the variation on gene structure by deletion is thought to alter light acceptance in chickens and positively affect egg production. Similarly, in *POPDC3* gene, the estimated copy number of CN and RIR was 2, which was very conserved among individuals. However, WL had an average copy number of 3. In the previous study, the same duplication was found in WL and associated with myometrium maturation, protein secretion, and eggshell formation [41]. Chicken breeds we analyzed are more likely to be subject to selective pressure for genes related to meat or egg production. Therefore, we thought that most of the genes with significant differences in ANOVA test would be ‘CN, RIR (meat) vs. WL’ or ‘WL, RIR (egg) vs. CN’. However, there were also many genes in the ‘CN, WL vs. RIR’ case. For example, *ADH6* gene, associated with glycolysis [82], showed duplication only in RIR, and *TLR2B* associated with immune response [83,84] showed deletion only in RIR. *PRSS2* gene showed deletion in both CN and WL breeds. However, when looking at the depth, the number of deleted copy numbers was different. Increasing the copy number of *PRSS2* gene, which encodes trypsinogen, is associated with chronic pancreatitis in humans [85]. However, the read mapping showed similar deletion patterns in the *PRSS2* gene as well as the overlapped gene, *TRBV6-5*. *TRBV* genes provide a variable component in the adaptive immune receptor biosynthesis, which is related to the beta chain of T-cell receptors [86]. The reason for the difference in copy number between breeds in *TRBV6-5* is similar to that of many CNVs detected due to a large number of immune-related genes in chromosome 16. This means that the immunological response can vary greatly between breeds [75]. We could identify duplication in the promoter region of *TPK1* and two deletions within the gene. Interestingly, duplication could only be found in CN and RIR, and deletion in WL. The known function of *TPK1* in livestock is to produce ATP by hydrolyzing thiamine diphosphate to thiamine when ATP is exhausted in the early stages of the postmortem period [87]. In addition, it has been reported that when *Escherichia coli* suffers from amino acid deficiency, it accumulates thiamin triphosphate, a higher phosphate derivative of thiamin, and acts as a signal related to adaptation to nutritional condition changes [88]. However, it is unclear what physiologically important function *TPK1* has in chickens. Therefore, further research is needed to determine the cause of copy number differentiation on *TPK1* between breeds.

## 5. Conclusions

In this study, whole-genome sequencing data of 65 chickens was collected from public databases to detect domesticated-specific CNVRs. By analyzing the function of annotated genes in the CNVR and genes that showed copy number differentiation between breeds, we investigated how genetic characteristics are linked to phenotypic characteristics of increased productivity. As a result, we have discovered that the genetic variation caused by artificial selection is effective on CNV level. The findings will be a great contribution to the understanding of poultry breeding and the development of economical traits along with other genomics studies on domesticated chickens.

## Figures and Tables

**Table 1:** Statistical summary of detected CNVs.

Breed	CNVs	Deletion	Size_Del (bp)	Duplication	Size_Dup (bp)
Red Jungle Fowl	714	584	20,377.4	130	16,019.1
Cornish	443	327	27,572.3	116	16,156.9
Rhode Island Red	471	346	26,778.9	125	16,394.3
White Leghorn	467	347	26,451.8	120	15,823

**Table 2:** Functional annotation of genes with significant differences in copy number between breeds (*p*-value < 0.05).

Category	ID	Term	Count	*p*-Value	Genes
BP	GO:2000251	Positive regulation of actin cytoskeleton Reorganization	2	1.88E-02	*NTRK3*, *HRAS*
BP	GO:0002224	Toll-like receptor signaling pathway	2	3.90E-02	*TLR2B*, *TLR2A*
BP	GO:0050852	T cell receptor signaling pathway	2	4.81E-02	*HRAS*, *CD247*
BP	GO:0060041	Retina development in camera-type eye	2	4.99E-02	*LPCAT1*, *RD3*
CC	GO:0031226	Intrinsic component of plasma membrane	2	1.58E-02	*TLR2B*, *TLR2A*
MF	GO:0016491	Oxidoreductase activity	5	3.44E-05	*LOC425137*, *LOC107053269*, *AKR1B1L*, *LOC772271*, *ADH6*
MF	GO:0004888	Transmembrane signaling receptor activity	3	2.57E-03	*CD247*, *TLR2B*, *TLR2A*
KEGG PATHWAY	gga01100	Metabolic pathways	7	3.10E-02	*TPK1*, *LOC425137*, *LPCAT1*, *AKR1B1L*, *ADH6*, *TWISTNB*, *ALDH3B1*
KEGG PATHWAY	gga00040	Pentose and glucuronate interconversions	2	4.25E-02	*LOC425137*, *AKR1B1L*

BP = biological process; CC = cellular components; MF = molecular function.

## Supplementary Materials

The following are available online at https://www.mdpi.com/2076-2615/9/10/809/s1, Figure S1: Estimated copy numbers of gene with different copy number between breeds and depth of coverage of gene region (expansion of Figure 4.), Table S1: Statistical summary of read mapping and detected CNVs, Table S2: CNVRs identified in domesticated chicken breeds, Table S3: Gene annotation of CNVRs, Table S4: Gene ontology analysis for identified CNVRs, Table S5: QTL annotation of CNVRs, Table S6: V_ST_ value of genes annotated in CNVRs, Table S7: ANOVA test of genes annotated in CNVRs.
