# Supplementary figures and images for: Identification of Copy Number Variation in Domestic Chicken Using Whole-Genome Sequencing Reveals Evidence of Selection in the Genome
Source: Animals (Basel). 2019 Oct 15;9(10):809. doi: 10.3390/ani9100809 (PMC6826909; doi:10.3390/ani9100809)

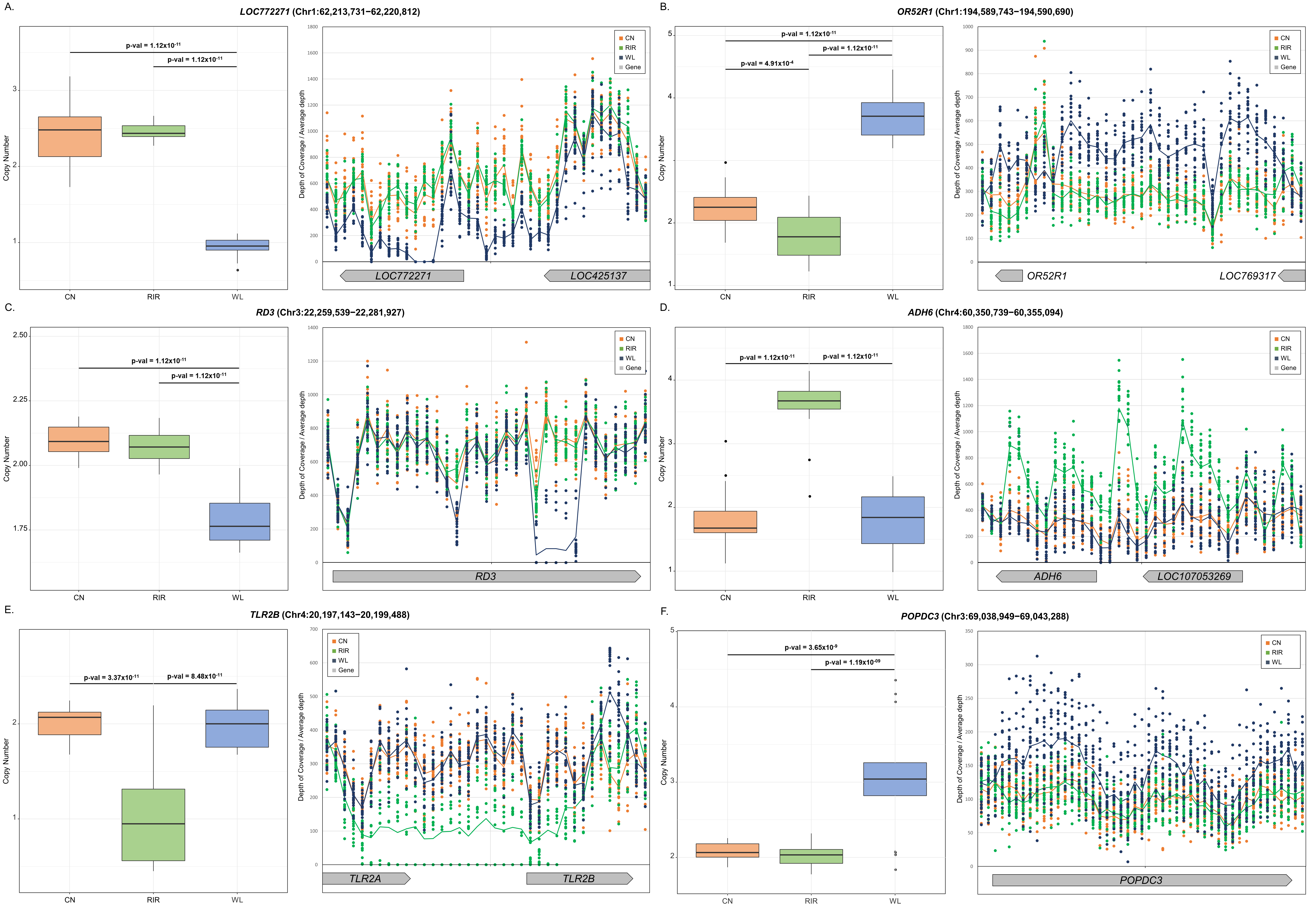

Supplement: Supplementary file 1 [file animals-09-00809-s001.zip › Supplementary Figure 1.tif]
